# Risks, Benefits, and Treatment Modalities of Menopausal Hormone Therapy: Current Concepts

**DOI:** 10.3389/fendo.2021.564781

**Published:** 2021-03-26

**Authors:** Jaya Mehta, Juliana M. Kling, JoAnn E. Manson

**Affiliations:** ^1^ Department of Internal Medicine, Mayo Clinic, Phoenix, AZ, United States; ^2^ Division of Women’s Health Internal Medicine, Mayo Clinic, Scottsdale, AZ, United States; ^3^ Department of Medicine, Brigham and Women’s Hospital and Harvard Medical School, Boston, MA, United States

**Keywords:** post-menopause, menopausal hormone therapy, estrogen, progesterone, hot flashes

## Abstract

Menopausal hormone therapy (HT) prescribing practices have evolved over the last few decades guided by the changing understanding of the treatment’s risks and benefits. Since the Women’s Health Initiative (WHI) trial results in 2002, including post-intervention analysis and cumulative 18-year follow up, it has become clear that the risks of HT are low for healthy women less than age 60 or within ten years from menopause. For those who are experiencing bothersome vasomotor symptoms, the benefits are likely to outweigh the risks in view of HT’s efficacy for symptom management. HT also has a role in preventing osteoporosis in appropriate candidates for treatment. A comprehensive overview of the types, routes, and formulations of currently available HT, as well as HT’s benefits and risks by outcomes of interest are provided to facilitate clinical decision making.

## Introduction

Menopausal hormone therapy (HT) prescribing practices have evolved over the last few decades guided by the changing understanding of the treatment’s risks and benefits. Prior to the Women’s Health Initiative (WHI) trial results in 2002, HT was generally accepted as appropriate and safe for treatment of menopausal symptoms, as well as for chronic disease prevention including cardiovascular disease (CVD) prevention (1–4). The unexpected findings of the WHI, which raised concerns about breast cancer and CVD risk in women taking oral conjugated equine estrogens (oCEE) and medroxyprogesterone acetate (MPA), led many women to stop taking, and many physicians to stop prescribing, HT (5). Sub-analysis of the WHI results by age group showed that the elevated risks identified for coronary heart disease (CHD) applied mainly to women who started HT after age 60 or a decade past menopause (5–8). In fact, the 18-year WHI follow up data showed no difference in cause specific mortality or all-cause mortality in women treated with HT vs. placebo and indicated favorable trends for all-cause mortality among the younger women treated with oCEE alone (9). Furthermore, subsequent randomized controlled trials such as the Kronos Early Estrogen Prevention Study (KEEPS) and the Early Versus Late Intervention Trial with Estradiol (ELITE) have continued to demonstrate a favorable safety profile of HT when started early in menopause (10, 11).

Symptoms are common during menopause with up to 70 - 80% of women experiencing vasomotor symptoms (hot flashes and/or night sweats).12 HT is the most effective treatment for the relief of vasomotor symptoms (VMS), and also reduces bone loss, fracture risk and can treat the genitourinary syndrome of menopause (GSM) when used locally (12, 13). Different types, formulations and routes of HT are available for use in women and may confer different risks and benefits. Given the improved understanding of the risks and benefits of HT, organizations across disciplines have published guidelines supporting the initiation of HT for symptomatic women who are within 10 years of menopause and under age 60 and without contraindications, such as breast cancer or existing cardiovascular disease. These organizations include the North American Menopause Society (NAMS), the American College of Obstetricians and Gynecologists (ACOG), and the Endocrine Society (13–15).

Overall, the recommendation is to use an individualized approach when treating symptomatic menopausal women with periodic reevaluation that includes the appropriate type, dose, formulation and route of administration to meet treatment goals for the duration needed. The following review aims to provide a practical tool for healthcare professionals caring for menopausal women by discussing in detail various types and formulations of HT available as well as the risks and benefits of HT for common outcomes of interest in clinical practice.

## Formulations and Route of Menopause HT

Various formulations and routes are available to allow for an individualized approach to menopause care including estrogens, progestogens, and tissue selective estrogen complex (TSEC). **Table 1** summarizes indications and contraindications for different formulations of HT.

**Table 1:** Indications and contraindications for menopausal hormone therapy (16, 17).

Formulation	Indications	Contraindications
**Estrogen-alone therapy** (women with hysterectomy) **Estrogen-progestogen therapy** (women with an intact uterus)	-Management of moderate to severe VMS -Prevention of osteoporosis in women unable to tolerate standard medications	*Absolute:* unexplained vaginal bleeding, liver disease, history of VTE, known blood clotting disorder, untreated HTN, history of breast, endometrial or other estrogen-dependent cancer, hypersensitivity to HT, history of CHD, stroke, or TIA *Relative:* High TG or gallbladder disease, elevated risk of breast cancer
**Tissue Selective Estrogen Complex (TSEC)**	-Same as above - Women with breast tenderness, increased breast density or uterine bleeding with EPT therapy	- Same as above
**Low-Dose Vaginal Estrogen Therapy**	-Treatment of genitourinary symptoms of menopause (i.e. vaginal dryness, dyspareunia)	*Absolute:* unexplained vaginal bleeding, known breast cancer, endometrial cancer or other estrogen-dependent cancer (unless reviewed/approved by the patient’s oncologist)

VMS, Vasomotor symptoms.

### Estrogen Therapy

Estrogen therapy alone is used for post-menopausal women who have undergone a hysterectomy (16). Estrogen formulations include human estrogens (17B-estradiol (E2), estrone (E1) and estriol (E3)), animal-derived estrogens (oCEE), and synthetic estrogens (ethinyl estradiol (EE)). The only FDA -approved, formulation of human estrogens is E2, which is the primary estrogen produced by the ovaries and most biologically active (16). oCEE is comprised of a mixture of estrogens derived from natural sources, such as the urine of pregnant mares (16).

### Routes

Estrogens are well absorbed through the gastrointestinal (GI) tract, skin and mucus membranes. Formulations are available as oral preparations, transdermal patches, sprays, gels, topical emulsion preparations, vaginal preparations, in combination with progesterone, and in a TSEC (16, 18). **Table 2** includes a comprehensive list of menopause HT available in the United States.

**Table 2:** Menopausal hormone therapy by formulation and route available in the United States (16).

Oral Estrogen							
Formulation			Brand Name			Dose (mg/d)	
Conjugated			Premarin			0.3, 0.45, 0.625, 0.9, 1.25	
Synthetic conjugated			Cenestin			0.3, 0.45, 0.625, 0.9, 1.25	
Esterfied			Menest			0.3, 0.625, 1.25, 2.5	
17β-Estradiol			Estrace, Gynodiol, Innofem, Generics			0.5, 1.0, 2.0	
Estradiol Acetate			Femtrace			0.45, 0.9, 1.8	
Estropipate			Ortho-Est, Ogen, Generics			0.625(0.75 estropipate), 1.25 (1.5), 2.5 (3.0), 5.0 (6.0)	
**Transdermal Estrogen**							
**Formulation**			**Brand Name**			**Dosage (mg)**	
17β-estradiol matrix patch			Alora Climar Esclim Estradot Fempatch Menostar Minivelle Vivelle Vivelle-Dot Generics			0.025, 0.05, 0.075, 0.1 twice/wk 0.025,0.0375, 0.05, 0.075, 0.1 once/wk 0.025,0.0375 0.05, 0.075, 0.1 twice/wk 0.025,0.0375 0.05, 0.075, 0.1 twice/wk 0.025 once/wk 0.014 once/wk 0.025, 0.0375 0.05, 0.075, 0.1 twice/wk 0.025,0.0375, 0.05, 0.075, 0.1 twice/wk 0.025,0.0375, 0.05, 0.075, 0.1 twice/wk 0.05, 0.1 once or twice/wk	
17β-estradiol reservoir patch			Estraderm			0.05, 0.1 twice/wk	
17β-estradiol transdermal gel			EstroGel Elestrin Divigel			0.035/d 0.0125/d 0.25, 0.5, 1.0 g/d	
17β-estradiol topical emulsion			Estrasorb			0.05/d (2 packets)	
17β-estradiol transdermal spray			Evamist			0.021/90 µL/d (up to 1.5/90 µL/d)	
**Combination Estrogen- Progestogen Therapy**							
**Regimen**		**Composition**			**Brand Name**		**Dosage (mg/d)**
Oral Continuous-Cyclic		CE (E)+ MPA (P)			Premphase		0.625 mg E+ 5.0 mg P (E alone x days 1-14, E+P days 15-28)
Oral Continuous-Combined		17β-estradiol (E)+ progesterone (P)			Bijuva		1 mg E +100 mg P
		CE (E)+ MPA (P)			Prempro		0.625 mg E+ 2.5 or 5 mg P 0.3 or 0.45 mg E + 1.5 mg P
		Ethinyl Estradiol (E) + NETA (P)			Femhrt		2.5 µg E + 0.5 mg P 5 µg+ 1 mg P
		17β-estradiol (E)+ NETA (P)			Activella		0.5 mg E + 0.1 mg P 1 mg E + 0.5 mg P
		17β-estradiol (E)+ drospirenone (P)			Angeliq		0.5 mg E + 0.25 mg P
Oral Intermittent-Combined		17β-estradiol (E)+ norgestimate (P)			Prefest		1 mg E + 0.09 mg P E alone x 3 days, E+P x 3 days, repeat
Transdermal Continuous-Combined		17β-estradiol (E)+ NETA (P)			CombiPatch		0.05 mg E + 0.14 mg P (9 cm^2^ patch, twice/wk)
		17β-estradiol (E)+ LNG (P)			Climara Pro		0.045 mg E + 0.015 mg P (22 cm^2^ patch, once/wk
**Tissue Selective Estrogen Complex (TSEC)**							
**Component**				**Brand Name**			**Dose**
oCEE (E) + Bazedoxifene (SERM)				Duavee; Duavive			0.45 mg E + 20 mg SERM, once daily
**Vaginal Estrogen Therapy**							
**Formulation**	**Composition**				**Brand Name**		**Dosage**
Vaginal Creams	17β-estradiol				Estrace		Initial 2-4 g/day for 1- 2 wk Maintenance: 1 g 1-3x/wk
	Conjugated Estrogens				Premarin		0.5 – 2g/d x 21days, off x 7 days
Vaginal Rings	17β-estradiol				Estring		2mg- releases 7.5 µg/d for 90 days
	Estradiol acetate				Femring		12.4 mg of 24.8 mg- releases 0.05 mg/d or 0.10 mg/d x 90 days*
Vaginal Tablet/Insert	Estradiol				Imvexxy		4 µg and 10µg Initial: 1 insert/d x 2 wk Maintenance: 1 tablet 2x/wk
	Estradiol hemihydrate				Vagifem		Initial: 1 insert/d x 2 wk Maintenance: 1 tablet 2x/wk

E, Estrogen; P, Progestogen; CE, Conjugated estrogens; MPA, Medroxyprogesterone acetate; NETA, Norethindrone acetate; LVG, Levonorgestrel; oCEE, Oral conjugated equine estrogens.

a, Oral products of synthetic estrogen mixtures, contain 75%-85% sodium estrone sulfate.

b, Oral form of estrone sulfate- solubilized and stabilized by piperazine.

*Leads to systemic levels – concomitant progestogen therapy recommended.

### Oral Estrogen Therapy

In the US, oral estrogen therapy in the most widely used formulation. Estradiol is converted to estrone during first pass metabolism such that estrone is the major hormone found in the circulation (16). Risks of oral estrogens largely stem from first pass metabolism through the liver and include increased production of coagulation factors and various inflammatory markers, hypertriglyceridemia, and elevated risk of venous thromboembolism (VTE), and gallstones (16).

### Transdermal and Topical Estrogens

Transdermal and topical estrogens bypass first pass metabolism so can be dosed lower than oral estrogen. Because of the avoidance of the first pass metabolism, they have less impact on triglycerides, coagulation factors and gallbladder disease (16, 18). Absorption varies based on how the patches and gels are applied. Transdermal therapy may not significantly increase VTE risk, in contrast to oral therapy, as seen in the Estrogen and Thromboembolism risk trial (19). A single nested case-control study showed stroke risk was not increased with transdermal HT, while it was with oral HT. KEEPS, the only randomized control trial (RCT) comparing oral and transdermal estrogen, was too small to allow a comparative analysis of risks of stroke, VTE, or other clinical events (16, 20).

Transdermal estrogen patches have a higher likelihood of causing skin irritation compared to other topical or oral formulations. Topical formulations including gels, sprays and emulsions may lead to a small amount of estradiol transferred if skin-to-skin contact is made within two hours of administration (16).

### Vaginal Estrogen Therapy

Low-dose vaginal estrogen therapy use is FDA approved to treat moderate-to-severe vaginal dryness and dyspareunia caused by GSM (13). It comes in multiple forms that are used vaginally including a cream, ring, tablet or capsule. Its primary mechanism is to locally treat postmenopausal vulvovaginal changes. It does not cause an increase in estrogen levels systemically above what is expected for a postmenopausal woman. Estrogen acetate vaginal rings (Femring) are the only vaginal estrogen used to treat vasomotor symptoms with systemic absorption (16). Apart from estrogen acetate vaginal rings, progesterone is not necessary for use with vaginal estrogen formulations given minimal absorption and low risk of endometrial cancer (16).

### Progestogen Therapy

Progestogen therapy is primarily used to avoid an increased risk of endometrial cancer for a woman on systemic estrogen, as unopposed estrogen thickens the uterine lining and increases risk of endometrial cancer. Progestogens protect against this endometrial thickening by decreasing estrogen receptors in target tissues and by inhibiting the luteinizing hormone surge that results in increased estrogen production from ovaries (16, 21). Progestogens aid in the conversion of estradiol to estrone in the endometrium by increasing 17B-hydroxysteroid dehydrogenase activity. Estrone’s weaker estrogen activity results in less endometrial stimulation (16).

Progestogen options include micronized progesterone and synthetic progestins, such as MPA or norenthindrone. Micronized progesterone is bioidentical to the hormone made endogenously and has efficient oral absorption. It can also be given vaginally, as was done in ELITE (10). Compared to natural progesterone, synthetic progestins have 10-100- fold greater activity (16).

Progestogens come in oral and transdermal forms. The most commonly used and widely studied formulation of progestogens in the United States (US) is the oral progestin MPA. Oral progesterone has mild sedating effects, micronized progesterone significantly decreases VMS and helps with sleep (22). Transdermal progesterones do not provide adequate endometrial protection so should not be used in combination therapy. The side effects of progestogen therapy include swelling and breast pain (more common with MPA), acne and hirsutism (more common with norethindrone), dizziness or fatigue, or adverse mood effects (23, 24).

### Combination Formulations

FDA-approved combined estrogen-progestogen formulations provide adequate dosing of progestogen for endometrial protection, and include continuous-cyclic estrogen- progestogen, continuous combined estrogen-progestogen therapy, and intermittent combined estrogen-progestogen therapy (16). Continuous- cyclic estrogen-progestogen therapy includes daily estrogen dosing with progestogen added cyclically for 12 to 14 days per month or in long cycle formulations, every 2 to 6 months for 14 days. Up to 80% of women on regimens that dose progestogens monthly have uterine bleeding with progestogen withdrawal. Those on the long cycle have less frequent withdrawal bleeding, though the uterine bleeding tends to be longer and heavier (16). Continuous-combined estrogen-progestogen therapy, the most commonly used formulation in North America, provides women estrogen and progestogen every day. These formulations prevent withdrawal bleeding that occurs with cyclic formulations (16). Lastly, intermittent-combined estrogen-progestogen includes daily estrogen with cycles of progestogen for 3 days and off progestogen for 3 days. Trials have shown intermittent progestogen dosing results in 80% amenorrhea rates after 1 year (16, 25).

While various options of combined estrogen progestogen therapy (EPT) are available, no evidence supports one regimen over the other. The Postmenopausal Estrogen/Progestin Interventions (PEPI) trial showed that, among women with a uterus on estrogen therapy alone, a 34% increased risk of endometrial hyperplasia, was observed, while those on combined therapy had a risk of 1% (23). Continuous- combined EPT showed no increased risk and possible protection against endometrial cancer compared to the general population (26).

Uterine bleeding with use of EPT oftentimes results from withdrawal bleeding after progestogen cessation or breakthrough bleeding. Forty percent of women on continuous- combined EPT will have break through bleeding in the first 3-6 months (27). Breakthrough bleeding occurs more frequently in women who begin HT within 1 year of menopause. Ultimately, most women on continuous EPT obtain amenorrhea (16). If women continue to bleed beyond 6 months of initiation of HT, they will need to undergo evaluation with an ultrasound and/or endometrial biopsy (28).

### Tissue Selective Estrogen Complex (TSEC)

TSECs pairs a selective estrogen-receptor modulator with estrogen. The FDA approved medication marketed as DuaVee combines 20 mg bazedoxifene, a selective estrogen-receptor modulator, with 0.45 mg oCEE for use in postmenopausal women with a uterus. It is used for moderate to severe VMS and for prevention of osteoporosis. Compared to placebo, TSEC had a similar profile in that it did not increase breast tenderness, breast density, or endometrial thickness; avoiding these conditions may be indications to use this therapy over other formulations. Amenorrhea occurs in more than 83% of users (29–32). It has not been studied regarding its ability to provide breast cancer risk reduction.

## Benefits and Risks of Menopause Hormone Therapy (HT)

Estrogen is the most effective treatment for VMS. Women with an intact uterus require combined progestogen therapy with estrogen for endometrial protection. For those without a uterus, estrogen alone can be used. Since the risk-benefit profile of HT treatment in symptomatic menopausal women is impacted by age, time since menopause and existing comorbidities, shared decision making is critical in determining what HT formulation and route to use and when discontinuation is appropriate (17). **Table 3** summarizes the evidence on risks and benefits of HT.

**Table 3:** Risk-benefit profile of menopausal hormone therapy.

Outcome/Symptom	Formulation	Benefit	Risk	Probable Benefit or Risk	Hazard Ratios	Ref
**Vasomotor Symptoms **(12, 13)	Estrogen (E)	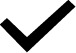				
	Estrogen+ Progestogen (E+P)	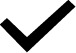				
**Osteoporosis **(7, 13)	E	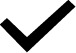				
	E+P	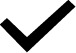				
**Coronary Heart Disease** (13, 33, 34–35–37)	E	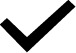 *	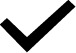 *	*Decreased risk in women <60, within 10 y of menopause *Increased risk in women many years past menopause		
	E+P	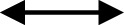	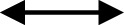			
**Stroke **(7)	E		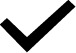			
	E+P		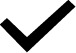			
**Type 2 Diabetes **(13)	E	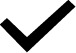				
	E+P	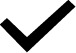				
**Venous Thromboembolism **(13, 36)	E		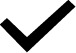			
	E+P		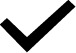			
**All-Cause Mortality **(13, 36)	E	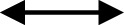		Trend toward decreased risk when started early in menopause and neutral or increased risk when started later in menopause.		
	E+P	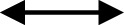				
**Breast Cancer **(7, 33, 38)	E	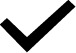				
	E+P		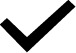			
**Endometrial Cancer** (7)	E					
	E+P	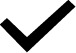				
**Colon Cancer **(7, 9, 39)	E	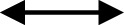				
	E+P	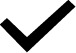				
**Fractures **(7, 13)	E	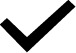				
	E+P	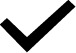				

### Cardiovascular

The relationship between HT and cardiovascular disease is complex. Prior to the WHI, observational studies had suggested that HT was associated with lower CVD incidence and all-cause mortality, comparing users and nonusers (40, 41). In the 1990s, the timing hypothesis, which postulates that initiating hormone therapy closer to menopause confers cardiovascular benefit, was first described in nonhuman primate studies. Clarkson and colleagues showed that estrogen therapy reduced the risk of coronary artery atherosclerosis in primates by 50-70% when started at the time of ovariectomy versus no benefit when started the human equivalent of many years after surgical menopause (42). Protective effects of HT on cardiovascular health stem from beneficial lipid modulation as well as estrogen’s favorable actions on the endothelium and vasculature (33, 43).

**Figure 1:**
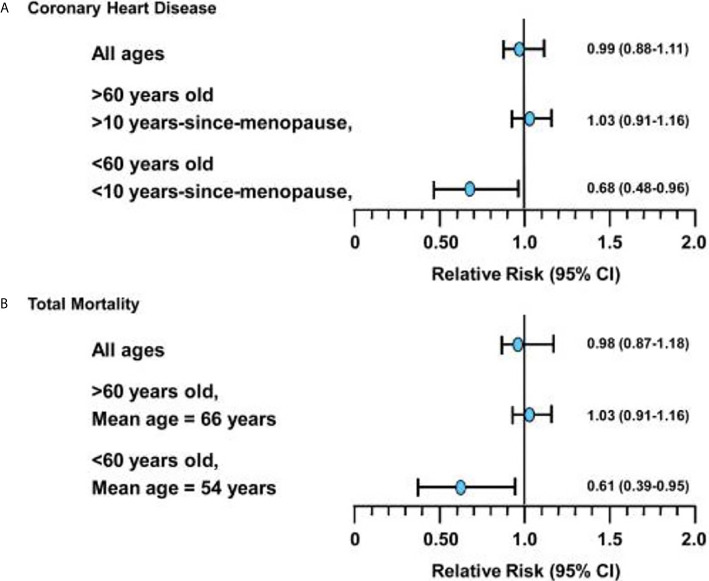
**(A)** Relative risks (and 95% confidence intervals) for coronary heart disease events associated with hormone replacement therapy from meta-analysis of 23 randomized controlled trials in 39,049 women (followed for 191,340 women-years). **(B)** Relative risks (and 95% confidence intervals) for total mortality associated with hormone replacement therapy from meta-analysis of 30 randomized controlled trials in 26,708 women (followed for 119,118 women-years). *Figure used with permission by John Wiley and Sons (License: 4838400238207) and from Dr. Howard Hodis from *Hodis HN, Mack WJ. The timing hypothesis: a paradigm shift in the primary prevention of coronary heart disease in women: part 1, comparison of therapeutic efficacy. J Am Geriatr Soc. 2013;61(6):1005-1010.*.

The WHI aimed to evaluate through an RCT the cardiovascular effects of HT in women, specifically assessing rates of CHD as the primary outcome. Unexpectedly, the WHI trial (which included women aged 50-79, mean age=63) showed an elevated risk of cardiovascular events in the oCEE + MPA treatment arm in all women (CHD HR =1.29, 95% CI 1.02 – 1.63, Stroke HR = 1.41, 95% CI 1.07-1.63) (24). Since 2002, WHI analyses stratified by age group, as well as observational studies and further RCTs including the Danish Osteoporosis Prevention Study (DOPS), KEEPS, ELITE have demonstrated that HT started in women <60 years or within 10 years of their final menstrual period (FMP) has neutral to beneficial effects on cardiovascular health (CVH) (20, 34, 35, 44) (**Figure 1**)

### Coronary Heart Disease and Cardiovascular Mortality

Based on data from the intervention phase of the WHI, women aged 50 to 79 years in the overall cohort on oCEE + MPA had increased risk of CHD, defined as non-fatal myocardial infarction and coronary death, compared to placebo, whereas women on oCEE alone had neutral CHD outcomes. Sub-analysis from the WHI showed that age made a difference in CHD outcomes, supporting the timing hypothesis. In the oCEE arm, women aged 50 to 59 trended toward decreased risk of CHD compared to women aged 70 to 79 (HR 0.60, 95% CI 0.35-1.04 vs HR 1.09, 95% CI 0.80-1.49) (20, 45). Follow up at 13 years supported prior data from the intervention phase of WHI that women who started oCEE alone at a younger age had lower CHD risk. In contrast, those randomized to oCEE + MPA at least 20 years after menopause had significantly higher risk of CHD compared to placebo (6, 7).

Importantly, cumulative follow up at 18 years showed no difference in cardiovascular mortality between the oCEE + MPA or oCEE group versus placebo for women age 50 to 79 years (HR:1.00, 95% CI: 0.92-1.08). Furthermore, there was no significant difference in CVD mortality when groups were stratified by age (7, 46). Potential explanations for why the intervention phase of the WHI showed unexpected cardiovascular effects include the older study population (mean age of 63 years), mean time since menopause of at least 12 years, numerous women with CV risk factors prior to enrollment, higher hormonal doses compared to subsequent studies and many participants who did not have vasomotor symptoms (33).

A Cochrane review in 2015 of RCTs of HT showed an overall reduction in risk of CHD and all-cause mortality in women who started HT within 10 years of menopause (36). Compared to older women, women in this group tend to have better baseline cardiovascular health with lower CVD risk and lower coronary artery calcium scores (13, 33, 36, 37). The safety of HT used early in menopause is further supported by the ELITE and KEEPS study. KEEPS was a 4 year randomized, double blinded, placebo-controlled clinical trial that randomized 729 women ages 42 to 58 years with a mean of 1.4 years from menopause to either oCEE 0.45 mg/d, transdermal estradiol 50 µg/d, with cyclical monthly progesterone or placebo. It assessed whether or not HT reduces progression of atherosclerosis when initiated early in menopause with a primary endpoint of carotid intima media thickness (CIMT) measurement. ELITE compared 643 women free of CVD either <6 years from menopause of >10 years past menopause, they were assigned to either oral estradiol 1 mg/day with or without micronized progesterone gel 45 mg/day x 12 days or to placebo for 6 years. The primary endpoint was CIMT measurement and secondary endpoints were change in neurocognitive function and coronary artery calcium (CAC) score (10). Both studies found that HT started in women who were early in menopause (less than 6 years) did not deleteriously impact subclinical atherosclerosis as measured by CIMT and, in ELITE, favorable effects of HT on CIMT were found in this group (47, 48).

DOPS, a large open-label study with no placebo arm, was started at the same time as the WHI and enrolled women between the ages of 45 and 58 who were randomized to HT with triphasic estradiol and norethisterone acetate, 2 mg estradiol per day or no treatment. Shierbeck, et al. used data from this trial to study a primary composite end point of death or admission to the hospital for heart failure and myocardial infarction. After 10 years of intervention, they found that those receiving HT had significantly reduced risk of the primary CVD composite endpoint compared to no treatment (HR: 0.48, 95%CI 0.26-0.87) (49).

**Summary:** HT has favorable or neutral effects on CHD risk when started in women younger than 60 years old or within 10 years of menopause, in the absence of contraindications.

#### Hormone type, formulation and route

The type, formulation and route of HT carry different side effects and risks. The WHI trial showed that unopposed oCEE has less adverse cardiovascular outcomes than an estrogen plus progestin formulation (33, 38, 50). Lower doses of estrogen (0.3 mg oCEE vs 0.625 mg oCEE) have comparable effects on major coronary events but may have a less adverse effect on the risk of stroke and possibly VTE (33). Transdermal estradiol also appears to have less adverse effects on VTE and stroke than oral estrogen formulations (51). Different types of progestogens also have varying effects; synthetic MPA is vasoconstrictive while natural progesterone and drospirenone cause vasodilation and lower blood pressure (33). Thus, when prescribing HT, it is important to take into account a woman’s cardiovascular health and risk factors, which may influence the HT formulation chosen.

### Stroke

The risk of ischemic stroke was higher in both the oCEE and oCEE + MPA groups during the WHI initial intervention study for women ages 50 to 79 years (HR: 1.35 [95%CI: 1.07 -1.76] and 1.37 [95%CI: 1.07-1.70]). No significant differences were noted when stratified by 10-year age groups (7). Cumulative follow up of WHI for 13 years (intervention plus post-intervention follow-up) showed an increased, but not statistically significant, risk in both treatment groups as well (HR: 1.16 [95%CI: 1.00-1.35] for oCEE+MPA and HR 1.15 [95%CI: 0.97-1.37] for oCEE alone), with no difference when stratified by age or time since menopause (7). Cumulative follow up for 18 years showed no significant effect on the risk of stroke mortality in the oCEE + MPA group (HR: 1.12 [95%CI: 0.91-1.38]) or in the oCEE alone arm versus placebo (HR: 0.98 [95%CI: 0.77-1.26]). In both the oCEE and oCEE+MPA trials, age did not significantly modify the results (9). The 2015 Cochrane review described no increased risk of ischemic stroke when HT was initiated less than 60 years of age and within 10 years of menopause (13, 36).

**Summary:** The findings for HT and stroke have been inconsistent, but systematic reviews suggest that stroke risk depends on age and time from menopause; women who initiate HT younger than 60 years of age or who are within 10 years of menopause onset do not appear to have an increased risk of stroke.

### Venous Thromboembolism

It has been well established that estrogen, especially oral estrogens, increase VTE risk (33, 52, 53). Therefore, it was not unexpected to find increased VTE events during the WHI. During the intervention phase of the WHI, the risks of pulmonary embolism (PE) and deep vein thrombosis (DVT) were significantly higher in the oCEE plus MPA group in the overall study population compared to placebo (HR:1.98 [95%CI:1.36-2.87] for PE, HR:1.87 [95%CI:1.37-2.54] for DVT). In the oCEE alone group, the risk of PE or DVT, was not statistically different from placebo in women ages 50 to 79 years (HR: 1.35, [95%CI: 0.89-2.05] for PE, but the HR was 1.48 (95% CI: 1.06-2.07) for DVT (7). During 13-year cumulative follow up (intervention plus post-intervention phases), no significant difference in VTE risk between either HT group or placebo was found (7).

A meta-analysis of 19 RCTs all using oral estrogens with or without a progestogen found that women who started HT fewer than 10 years after menopause and younger than 60 showed that it increased the risk of VTE compared to placebo, without differences by age group (RR: 1.74; 95% CI: 1.11-2.73) (13, 36). Formulation and route of HT has been shown to affect VTE risk. VTE risk is lower with low doses of oral estrogen, micronized progesterone rather than synthetic progestins and with transdermal formulations of HT rather than oral (13, 33, 54–56). A nested case control study done in the UK found postmenopausal women who took oral estrogen alone had a lower risk of VTE with oral estradiol compared to oCEE (OR:0.85, 95% CI 0.76-0.95). Compared to oCEE + MPA, oral estradiol with either MPA, dydrogesterone or norethisterone was associated with a lower risk of VTE (OR: 0.68 [95%CI: 0.51-0.91], OR: 0.56 [95%CI: 0.45 -0.69] and OR: 0.80 [95%CI: 0.71-0.89], respectively). Transdermal HT preparations were not associated with increased risk of VTE (OR: 0.93 [95%CI: 0.87-1.01]) (51).

**Summary:** Risk of VTE differs based on HT formulation. Oral HT increases the risk of VTE, whereas transdermal HT does not appear to increase the risk of VTE.

### Heart Failure

Estrogen deficiency is thought to increase risk of heart failure with preserved ejection fraction due to an increase in cardiac mass and association with diastolic dysfunction (35). Small observational studies indicated HT was associated with lower mortality in menopausal women with heart failure (57). An analysis of 644 women with heart failure included in HERS found that there was no difference in all-cause mortality compared to placebo (HR: 1.0, 95%CI: 0.7-1.4) (57). Similarly, a recent study assessed the effects of HT on incidence of heart failure and its subtypes, as well as differences by age using data from the WHI. Overall, there was no significant effect of HT on the risk of heart failure with or without reduced ejection fraction, but younger women assigned to oCEE showed a signal for benefit for the former (58).

**Summary:** Overall, HT does not seem to alter the risk of heart failure with or without reduced ejection fraction, or the risk of heart-failure-related mortality.

### Type 2 Diabetes

Oral and transdermal estrogen use have both been shown to lower blood glucose and improve insulin sensitivity; while oral estrogen has a stronger effect (59). Estrogen is thought to improve glucose metabolism by acting on estrogen receptors that allow for improved insulin sensitivity and insulin secretion (59).

Women on oCEE + MPA in the WHI had a statistically significant reduced risk of type 2 diabetes mellitus (T2DM) compared to placebo (HR, 0.81: 95% CI, 0.70-0.94). Women in the oCEE-alone arm also showed this benefit (HR, 0.86, 95% CI: 0.76-0.98) (13). The Heart and Estrogen-Progestin Study (HERS), a randomized, blinded, placebo-controlled study that evaluated cardiovascular outcomes in post-menopausal women younger than 80 years with established coronary disease (0.625 oCEE + 2.5 mg MPA daily vs placebo), found that combined estrogen and progestin reduced the risk of T2DM (HR:0.65, 95% CI, 0.64-0.80) (60, 61). A meta-analysis of 18 studies found that HT use showed a significant reduction in insulin resistance among non-diabetic women and a 30% relative risk reduction in developing T2DM (62). Another meta-analysis of 16 studies showed similar findings with a reduction in incidence of diabetes in those on HT compared to placebo (OR: 0.61, 95% CI:0.55-0.68) and significantly lower fasting blood glucose and HbA1c compared to placebo (63).

**Summary:** HT reduces the risk of T2DM, lowers fasting blood glucose and HbA1c and improves insulin sensitivity.

### Metabolic Syndrome

Weight gain and central weight re-distribution is common for women post-menopause (64–66). Disruption of estradiol signaling after menopause, either naturally or surgically, may cause increased and accelerated fat accumulation in the abdominal area, resulting in increased insulin resistance, dyslipidemia, hypertension and cardiovascular disease (13, 65, 67). HT appears to be weight neutral when used in menopause, although it does impact weight distribution with an increase in lean body mass and decrease in visceral fat (62, 68). During the first year of the WHI, women in the oCEE plus MPA arm, compared to placebo, had a significant decrease in body mass index (BMI) and waist circumference (13). A meta-analysis reviewing 9 RCTs found that HT increased lean body mass in non-diabetic patients, and reduced waist circumference and abdominal fat (62).

After menopause it’s not uncommon to see increases in total cholesterol, increases in low-density lipoprotein (LDL), and increases in lipoprotein (a) (Lp(a)) (43). A meta-analysis that reviewed 61 studies found that HT increases high-density lipoprotein (HDL) and decreases LDL, LDL/HDL ratio and Lp(a) (62). The beneficial effects on LDL/HDL ratios are greater with oral formulations rather than transdermal. OCEE contributes to better lipid profiles when compared to oral esterified estrogens (62).

**Summary:** HT tends to be weight neutral in post-menopausal women, although it may favorably affect body composition, including decreasing visceral fat, increasing lean body mass, and improving the lipid profile. Specific effects, however, vary by HT formulation, regimen, and route of delivery.

### Dementia

Women suffer from Alzheimer’s disease (AD), the most common form of dementia, at a higher incidence than men. While this may be due to the increased life-span of women, it is also possible that sex-specific factors such as reductions in estrogen and organizational effects of sex steroid hormones in the female brain early in development confer increased risk and influence incidence differences (69, 70). The effects of HT on dementia and cognition in post-menopausal women have been evaluated (71). Prior to the WHI, multiple observational studies suggested that use of HT reduced or delayed the risk of AD (72–74). Estrogens have been shown to be neuroprotective in animal studies (69). They promote cholinergic and serotonergic activity in specific areas of the brain, maintains neural circuits and prevent cerebral ischemia (71).

In the WHI, women older than 65 years were enrolled in the WHI Memory Study (WHIMS). Women in the oCEE + MPA arm had double the risk of dementia compared to women assigned placebo (oCEE+MPA HR 2.01 (95% CI: 1.19-3.42); oCEE alone HR 1.47 (95% CI, 0.85- 2.52) (7, 13). The Women’s Health Initiative Memory Study of Younger Women (WHIMSY) included women aged 50-54 and found women on oCEE with or without MPA had neutral effects on cognitive function at an average of 7.2 years of follow up (75). A nationwide case control study done in Finland compared HT use in 84,739 post-menopausal women with and without a diagnosis of AD without controlling for many medical confounders. Results showed an increased risk of 9%-17% of AD in women on either estrogen-progesterone therapy or estrogen therapy alone (OR: 1.17, 95%CI: 1.13-1.21 and OR: 1.09, 95%CI: 1.05 -1.14, respectively). The study found an increased risk of AD with 10 or more years of HT use, shorter duration of use was not associated with increased risk of AD (71). Given the observational nature of the study, and the fact that many important medical confounders were not accounted for, additional data are needed prior to changing clinical practice.

Shao et al. examined whether or not the timing or type of HT played a role in risk of AD in 1,768 women in the population-based Cache County Study. Results showed that women who started HT within 5 years of menopause had a 30% reduced risk of AD (95% CI: 0.49-0.99). Those who started HT 5 or more years after menopause had increased rates with a similar HR as the WHI memory study (HR: 1.93, 95% CI:0.93-3.96) (76). Similarly, ELITE found that oral estradiol did not impact verbal memory, executive functions, or global cognition in women who started HT 6 years within menopause or 10 years or more after menopause (77). KEEPS had similar neutral findings in recently menopausal women (78). Thus, similar to cardiovascular health, it is possible that the timing hypothesis may also apply to HT’s effects on cognition. KEEPS had similar neutral findings (78).

**Summary:** HT appears to increase the risk of AD when initiated in older women or those more distant from menopause onset. When initiated in early menopause, however, HT appears to have a neutral effect on cognitive function.

### Select Oncologic Topics

#### Breast Cancer

Both observational studies prior to the WHI and the WHI suggested increased breast cancer risk in HT users (38, 79). The risk differed based on formulation and route of HT used, as well as if a progestogen was required (13). In the WHI, the risk of breast cancer was significantly increased for those on oCEE + MPA compared to placebo during the intervention phase (HR: 1.24, 95%CI: 1.01-1.53). The risk corresponded to 9 more cases of breast cancer per 10,000 women who were on oCEE+ MPA for 5 years or more (33, 38).

During 13-year cumulative follow-up, women randomized to oCEE + MPA continued to show statistically significant risk of invasive breast cancer compared to women assigned to placebo (HR: 1.28, 95% CI: 1.11-1.48). The absolute risk of breast cancer is considered rare with <1 additional case/1,000 person years of use, which is less than the risk associated with drinking two glasses of wine per day and similar to the risk seen with obesity or a sedentary lifestyle (80, 81).

Those on oCEE alone did not show an increased risk, and in fact showed a nonsignificant reduction in breast cancer risk after an average of 7.2 years of randomization. At 11.8 years, those on oCEE vs placebo had a statistically significant reduction in overall breast cancer (HR: 0.77, 95% CI: 0.62-0.95) and in deaths from breast cancer (HR: 0.37, 95% CI: 0.13-0.91) (82). At 13 year cumulative follow up, a statistically significant reduction in breast cancer persisted (HR: 0.79, 95% CI, 0.65-0.97) (7). At 18 year follow up, a statistically significant reduction in breast cancer mortality persisted in the oCEE alone group (HR: 0.55, 95%CI, 0.33-0.92, p=0.02) (46).

Breast cancer risk in the setting of HT is likely variable based on formulation of HT. Transdermal estradiol alone may have less of a protective effect on breast cancer risk than oral oCEE alone due to different effects on the breast tissue; however, prospective randomized controlled trials have not been done to compare them directly and sub-analysis of the Million Women Study found no statistical differences between the different formulations (33, 83). Additionally, the duration of HT treatment likely impacts breast cancer risk. In the WHI, it was after 5.2 years that risk increased in the oCEE + MPA arm. Five or more years after stopping HT, the risk elevations persisted (33, 79, 83).

**Summary:** Breast cancer risk varies based on HT formulation and duration of use. oCEE +MPA increases risk of breast cancer but not breast cancer mortality. In contrast, oCEE alone reduces the risk of breast cancer and breast cancer mortality.

#### Colorectal Cancer

Post-menopausal women have been shown to have a 1.5 times higher risk of colon cancer than pre-menopausal women of the same age (84). Prior to the WHI, case control studies suggested a reduction of colon cancer in post-menopausal women on HT (84). During the intervention phase of the WHI, women on oCEE plus MPA had a statistically significant lower risk of colon cancer than those receiving placebo (HR: 0.62, 95% CI: 0.43-0.89) (7, 85). Observational studies support this finding, especially when HT is started close to menopause (13, 86). Incidence of colon cancer was similar for oCEE and placebo in the oCEE-alone trial during the intervention phase (7). Findings showed no statistically significant difference from placebo in the two trials for incidence of colorectal cancer at 13 years or colorectal cancer mortality at 18 year follow up (HR: 0.80 [95%CI: 0.63-1.01) for oCEE+MPA, HR: 1.13 [95%CI: 0.85-1.51] for oCEE at 13 year follow up, and HR: 1.10 [95%CI: 0.82-1.46] pooled at 18 years follow up) (7, 9).

Observational studies since the WHI have shown protective effects of recent and current HT use on colorectal cancer with reduction in incidence and mortality from colorectal cancer (33). An observational study done in 2017 found a reduction in risk of colorectal cancer for those who had ever-used HT (HR: 0.90, [95% CI: 0.84-0.95]) (87). Another in 2018 by Symer et al. found reduction in risk and in death from colorectal cancer for current users of HT compared to never users (HR:0.81, p=0.005 and HR:0.63 and p=0.002, respectively) (39). Additional research is needed to clarify the relationship of HT, especially its formulation and timing of use, and colon cancer risk.

**Summary**: While more research is needed, initial data suggests oCEE + MPA lowers the risk of colon cancer while oCEE alone does not.

#### Endometrial Outcomes

Women on unopposed estrogen therapy are at risk for endometrial hyperplasia and endometrial cancer, which is the 4^th^ most common cancer in women in the US and hormone dependent in 90% of cases (33, 88, 89). The WHI and subsequent studies looked at the relationship of HT and risk of endometrial cancer. During the intervention phase of the WHI, those on oCEE plus MPA did not show a statistically significant difference in incidence of endometrial cancer compared to placebo. Post-intervention and cumulative 13-year data, however, showed a statistically significant reduced risk of endometrial cancer with oCEE + MPA compared to placebo; post-intervention (HR: 0.58, 95% CI: 0.40 – 0.86) and cumulative (HR: 0.67, 95% CI 0.49-0.91) (7).

Interestingly, after the WHI data came out there was a decline in HT prescriptions which correlated with an increased incidence of endometrial cancer. Watchel, et al. found that the rate in 2012 was 1.46 times higher than the rate in 2001. This supported the data found in the WHI of protective effects found in the WHI of combined HT (33, 90). A systematic review of 28 studies published in 2016 supported increased risk of endometrial cancer with unopposed estrogen use even when use was less than five years, with persistence of risk for more than 10 years (89, 91, 92). The review studied differences in formulations of combined HT and found that continuous combined estrogen progestogen therapy may have protective effects; however, risk may be increased with current micronized progesterone use and sequential norethisterone acetate use (89, 93, 94).

A prospective, non-randomized study found that women with stage I-II endometrial cancer did not have an increased risk of recurrence compared with age matched controls when treated with oCEE+MPA (88, 95). An RCT found this to be true for women who had early stage disease, had undergone hysterectomy, and were treated with estrogen therapy alone (88, 96). Given these data, consideration can be given to HT treatment in women who have bothersome VMS after early surgical menopause related to early stage endometrial cancer (13).

**Summary:** Although HT should be avoided in women with a history of estrogen-sensitive cancers, it can be considered for VMS management among women with a history of early-stage endometrial cancer.

#### Total Cancer and Total Cancer Mortality

During the intervention phase of the WHI, neither HT intervention was associated with increased total cancer incidence or total cancer mortality. When stratified by age, women 50 to 59 years in the oCEE arm had a lower incidence of total invasive cancer (HR: 0.80, 95% CI: 0.64-0.99) (7). Total cancer mortality in the overall cohort was similar between both intervention groups and placebo at 18 year follow up (HR: 1.06 [95%CI:0.95-1.18] for oCEE+MPA and HR: 0.99 [95%CI:0.86-1.13] for oCEE alone) (9).

**Summary:** At long term follow up, the incidence of total cancer mortality did not differ between the HT and placebo groups.

### Bone Health

Osteoporosis affects 10 million Americans and is projected to increase by 50% by 2025 (97). Post-menopausal women are at high risk and make up a majority of those with osteoporosis. Estrogen induces osteoclast apoptosis- a protective mechanism that declines after menopause, resulting in higher osteoporosis and fracture risk (98). HT inhibits osteoclast activity and prevents bone loss in postmenopausal women (13). HT is FDA approved for prevention of bone loss and osteoporosis in post-menopausal women (13).

#### Hip Fracture

Both oCEE + MPA and oCEE alone reduced risk of hip fractures by 33% compared to placebo during the intervention phase of the WHI (HR: 0.67 for both groups [95%CI: 0.47-0.95] and [95%CI:0.46-0.96], respectively) (7). Cumulative 13-year follow up showed attenuated effects of oCEE on reducing hip fractures, however those on oCEE plus MPA continued to have a statistically significant reduced risk of fracture compared to placebo (HR, 0.81, 95%CI: 0.68-0.97) (7). In comparison, bisphosphonate therapy with alendronate reduces spine and hip fracture by 50% over three years (98).

**Summary:** Both oCEE + MPA and oCEE alone significantly reduce the risk of hip fracture, although these benefits may dissipate over time after stopping HT.

#### Vertebral Fracture

The risk of vertebral fractures was significantly lower during the intervention phase for both oCEE plus MPA and oCEE compared to placebo (HR: 0.68, [95% CI 0.48-0.96] and HR: 0.64, [95% CI, 0.44-0.93]) (7). The reduction of vertebral fractures from bisphosphonate therapy ranges from 41%-70% (98).

**Summary**: HT reduces the risk of vertebral fractures.

#### All Fractures

Intervention data showed a significant risk reduction of all fractures in both HT groups compared to placebo (HR: 0.76, [95% CI: 0.69-0.83] and HR: 0.72, [95% CI: 0.64-0.80]) (7). Pooled results from 5 studies showed a significant reduction of fractures in women on combined HT (RR: 0.80 [95%CI: 0.68-0.94]) (99). For comparison, bisphosphonate use is associated with a 45% lower chance of vertebral fractures and 27% lower chance of non-vertebral fractures (OR 0.55, 95%CI: 0.44 to 0.69 and OR: 0.73, 95%CI: 0.67 – 0.81, respectively) (100). A meta-analysis by MacLean, et al. compared 6 studies and found there was no statistically significant difference between bisphosphonates and estrogen in preventing fractures (101).

**Summary:** HT reduces risk of all fractures. A difference in preventing fractures has not been found between bisphosphonates and estrogen.

#### All-Cause Mortality

All-cause mortality was not different between either HT group vs placebo during the intervention phase of WHI or during 13 year cumulative follow up (7). The cumulative follow up at 18 years showed no increased risk of all-cause mortality in either the oCEE or the oCEE plus MPA groups (HR: 0.94, [95% CI, 0.96-1.08] and HR: 1.02 [95% CI, 0.96-1.08], respectively). When stratified by age, younger women (age 50-59) had more favorable results for all-cause mortality than older women during the intervention phase of the oCEE trial and pooled trial (p-values for trend by age = 0.04 and 0.01, respectively), and a mortality risk reduction persisted over 18 years of cumulative follow-up among the younger women in the oCEE trial (9). Both a Cochrane review of RCT data and a meta-analysis of studies among women early in menopause found women initiating HT within 10 years of menopause or younger than 60 years of age had a significant reduction in all-cause mortality (RR: 0.70 [95% CI, 0.52-0.95]), but older women did not have a mortality reduction (13, 36).

**Summary:** The effect of HT on all-cause mortality varies by age, with a trend toward reduction in mortality among younger women but not among older women distant from menopause onset.

### Special Populations

#### Early Menopause

Early menopause is associated with numerous long-term health consequences including increased risks of osteoporosis, cognition and mood changes, heart disease, stroke, Parkinson disease, ophthalmic disorders and early mortality (13, 102). Those who undergo early menopause, either naturally or surgically, are deprived of estrogen at an early age, which is likely the underlying cause for poorer health outcomes long term. It is also the reason why HT should be considered for these women at least until the average age of menopause, which may help to mitigate the health risks associated with early menopause (13). Professional societies recommend this approach, in the absence of contraindications.

#### Bilateral Oophorectomy

Women undergoing hysterectomy with bilateral oophorectomy have a reduced risk of ovarian and breast cancer but do face adverse effects if menopause is induced prematurely (103). Bilateral oophorectomy results in an abrupt loss of ovarian produced estrogen, progesterone and testosterone. The health effects associated with the loss of these hormones can significantly affect a woman’s health and quality of life. Estrogen therapy in women with history of early bilateral salpingo-oopherectomy (BSO) has been associated with a reduction in bone disorders, mood changes, cognitive dysfunction, coronary heart disease and all-cause mortality, as well as improved sexual health (13, 103).

A recent sub analysis of the WHI estrogen- alone arm evaluated women with prior hysterectomy with or without BSO to assess effects of HT on women depending on BSO status. Both during the intervention phase and at cumulative 18 year follow up, there was no difference in HT’s effects on CHD, breast cancer, all-cause mortality or a global index score (including the aforementioned measures plus stroke, PE, colorectal cancer and hip fracture) between women with or without BSO. Global index HRs during the intervention phase generally increased (were more adverse) as age increased: 0.85 (95%CI: 0.54-1.34), 0.94 (95%CI: 0.74-1.19) and 1.42 (95% CI: 1.09-1.86) for respective 10-year age groups. During an 18 year cumulative follow up, younger women in the oCEE group had significantly reduced all-cause mortality compared to older women (HR:0.68 [95%CI:0.48-0.96] for 50-59 y, HR: 0.88 [95%CI:0.74-1.05] for 60-69 y and HR: 1.02 [95%CI:0.86-1.21] for 70-79 y). Those without BSO did not show a difference when stratified by age during the intervention phase and at cumulative follow up (103). Thus, in women aged 50 to 59 with prior hysterectomy and BSO, estrogen therapy conferred significant benefit.

#### Women With a History of Breast Cancer

Systemic HT is not recommended in women with active breast cancer or a history of breast cancer due to an increased risk of recurrence (13, 104–106). Instead, women with a history of breast cancer may choose non-hormonal options to treat bothersome VMS, such as antidepressants and gabapentinoids (107). Women may consider low-dose vaginal estrogen therapy for bothersome refractory vulvovaginal symptoms related to the genitourinary syndrome of menopause (GSM) after a discussion with their oncologist and primary doctor (13, 108). For women who are taking aromatase inhibitors, low-dose vaginal estrogen therapy should not be used, thus avoiding an increase in circulating estrogen levels (108). Non-hormonal treatments including lubricants with intercourse, or regular vaginal moisturizer therapy can instead be considered.

#### Persistent Symptoms

Many women continue to report vasomotor symptoms beyond age 60, with over 40% of women aged 60 - 65 reporting moderate to severe VMS (13, 109). For those women with persistent bothersome symptoms, a discussion about benefits of HT relief vs risks associated with continuing HT beyond age 60 is warranted. Extending HT use with the lowest effective dose is acceptable under some circumstances, and the decision to discontinue should not solely be determined based on a woman’s age. This recommendation is supported by the North American Menopause Society Position Statement from 2015 and 2017 (13, 110).

## Conclusion

HT is the most effective treatment for the vasomotor symptoms of menopause, which are common and can adversely impact a woman’s life. It is clear now that the safety of HT depends on a woman’s age and duration of time since menopause, such that the benefits tend to outweigh the risks in healthy women less than age 60 or within ten years from menopause. There are various types, formulations and routes of HT available to women so that their healthcare practitioner can tailor and individualize their treatment to manage their symptoms. Guidelines from NAMS, ACOG and the Endocrine Society as well MenoPro app, a free mobile app and decision-support tool from NAMS, provide assistance to practitioners treating menopausal women.

## Author Contributions

JM, JK, and JEM all contributed to the literature review, drafting and editing of the manuscript. All authors contributed to the article and approved the submitted version.

## Conflict of Interest

The authors declare that the research was conducted in the absence of any commercial or financial relationships that could be construed as a potential conflict of interest.
